# Heterogeneity in the guidelines for the management of diabetic foot disease in the Caribbean

**DOI:** 10.1371/journal.pgph.0000446

**Published:** 2022-05-13

**Authors:** Bauer E. Sumpio, Simone McConnie, Dale Maharaj

**Affiliations:** 1 Department of Vascular Surgery, Yale University School of Medicine, New Haven, Connecticut, United States of America; 2 Comfeet Foot Care Clinic, Christ Church, Barbados; 3 Caribbean Vascular & Vein Clinic, Port of Spain, Trinidad and Tobago; Bangladesh University of Health Sciences, BANGLADESH

## Abstract

The prevalence of diabetes mellitus, diabetic foot (DF) disease and, as a result, lower extremity amputation rates remain high in the Caribbean. This study was undertaken to determine whether Caribbean countries have designated individuals that monitor DF disease and whether there are DF protocols consistent with the International Working Group on the Diabetic Foot (IWGDF) guidance documents. Relevant DF health care personnel(s) from the CARICOM and Dutch Caribbean countries were called or sent questionnaires regarding the presence of structured programs to monitor and manage DF problems in the population. All 25 countries (100%) responded. 81% of respondents could not identify any Ministry, Hospital or individual initiatives that monitored the DF. Only 9 (36%) countries had any guidelines in place. Only 3 countries with guidelines in place utilized IWGDF guidelines. Only 6 (24%) countries had podiatrists and 10 (40%) had vascular surgery availability. 7 (28%) countries had the components for a multidisciplinary team. The presence or the appointment of a designated individual and/or a multidisciplinary approach within the countries for DF disease was absent in the majority of respondent countries. Only a minority of countries implemented DF guidelines or had expertise available to organize a DF multidisciplinary team. Vascular surgery and podiatric care were noticeably deficient. These may be critical factors in the variability and reduced success in implementation of strategies for managing DF problems and subsequent amputations amongst these Caribbean countries.

## Introduction

Lower extremity (LE) ulceration is prevalent throughout the world and poses a major threat to limb integrity and life. Foot ulcers occur in up to 25 percent of patients with diabetes and precede more than 8 in 10 non-traumatic amputations [1]. In 2014, the World Health Organization (WHO) estimated that 422 million people were diagnosed with diabetes with worldwide prevalence rates reaching nearly 9.3% [2]. This is a major *global* public health problem and it is estimated that a major amputation occurs every 20 secs world-wide [3]. In the Caribbean, the overall prevalence of diabetes mellitus is estimated to be approximately 9% and is responsible for 13.8% of all deaths among adults in the region [4]. The prevalence of diabetic foot (DF) disease is high in the Caribbean and unfortunately, some countries have been labeled as “amputation capitals of the world” because of their high LE amputation rates [5]. The loss of limb as a result of diabetes is especially harsh in the Caribbean because lower limb prosthetics are not routinely available [6–8].

The socio-economic impact of diabetes is profound. In 2015 the estimated annual global direct and indirect costs of diabetes was approximately US$1.3 trillion, with one in five diabetes dollars spent on lower extremity care [9]. In Latin America and the Caribbean, it was estimated that the cost of diabetes was US$135 billion in 2015 [10]. This burden included loss of productivity due to mortality and disability, as well as direct medical costs caused by treating diabetes and its long–term complications. The indirect cost of diabetes was US$826 million for the Caribbean [11]. In Trinidad and Tobago alone it was US$85 million [11]. Therefore, preventing foot ulcerations and/or LE amputations is critical from both medical, economical and socio-economical standpoints.

The pathophysiologic mechanisms underlying DF disease are multi-factorial and include neuropathy, infection, ischemia, abnormal foot structure and biomechanics [1, 12]. It is, therefore, not surprising that the management of the DF is a complex clinical problem requiring an interdisciplinary approach [13]. Implementation of evidence‐based management of DF disease has been shown to significantly reduce hospitalization, LE amputation, disability, mortality, and cost burdens [13, 14]. Despite public health and care-giver DF initiatives, there does not appear to be any significant improvement in the number of LE amputations in most of the Caribbean [6, 8, 15].

Many developed nations create their own DF disease guidelines, or adapt those issued by the International Working Group on the Diabetic Foot (IWGDF) that has developed and distributed evidence‐based, guidance documents developed through consensus of experts in clinical and research DF disease [16]. The 2015 IWGDF guidance documents provide recommendations on prevention, appropriate footwear and offloading, management of vascular disease, infections, wound healing and the need for use of a multidisciplinary approach. DFoot International is the implementation group of the IWGDF and is organized around seven regions of which North America and the Caribbean (NAC) is one [17]. D-Foot International promotes the global profile of DF prevention and care through awareness, guidance, education, research, and professional development. They promote training of healthcare professionals, in the implementation of appropriate strategies and building of teams in the prevention (through early screening) and management of DF problems effectively and provide strategies to develop foot services. Given the historical accounts of the dire state of DF management in the Caribbean, this study aimed to determine whether the Caribbean countries have protocols in place to monitor and manage DF disease consistent with the IWGDF guidance documents. We specifically queried whether there were responsible institutions or individuals in the country that were designated with this responsibility.

## Materials and methods

A questionnaire modeled from a previous study comparing diabetic foot guideline utilization in Western Pacific nations [18] was sent to 15 CARICOM member countries (Antigua and Barbuda, Bahamas, Barbados, Belize, Dominica, Grenada, Guyana, Haiti, Jamaica, Montserrat, St. Lucia, St. Kitts and Nevis, St. Vincent and the Grenadines, Suriname and Trinidad and Tobago), 5 CARICOM Associate member countries (Anguilla, Bermuda, British Virgin Island, Cayman Island and Turks and Caicos) [2], the Dominican Republic as well as the Dutch Caribbean countries (Aruba, Bonaire, Curacao and Sint Marteen). The known national representatives of the International Diabetic Foot (IDF) and DFoot organizations of these countries were contacted by email and/or phone and invited to participate in the survey. In countries that did not have any representation, we contacted the Ministry of Health (MOH), the national diabetes association or the national medical association.

The survey (Table 1) asked whether DF guidelines existed and whether the MOH, National Diabetes Association, Public Hospital, Medical University or Clinical Departments were responsible for implementation or involved in their enforcement. If guidelines existed, we sought to determine what they were and whether they followed IWGDF protocols. Information on the numbers of the relevant healthcare professionals known to be essential for the DF multidisciplinary team (general surgeons, orthopedic surgeons, vascular surgeons, podiatrists, infectious disease specialists, endocrinologists, wound care specialists and wound care nurses) was also queried.

**Table 1:** 

Questionaire
Do you know if your country has guidelines for the management of the Diabetic Foot?—Y/N Could you provide it?—Y/N
If you do not have it, we would like to determine if one exists in your country Does your country have a Diabetes Association- Y/N Do they have guidelines for the management of the Diabetic Foot?—Y/N Does your country have a representative on the DFoot International?—Y/N Who is that person if not you? Does your country have a Medical (or other Paramedical) Association- Y/N Do they have guidelines for the management of the Diabetic Foot?—Y/N Does your country have a University Department of Surgery/Medicine/Primary Care- Y/N Do they teach guidelines for the management of the Diabetic Foot?—Y/N Does your country have a Ministry of Health- Y/N Is there a designated individual that deals with diabetes and/or diabetic foot- Y/N Do they have guidelines for the management of the Diabetic Foot?—Y/N
Please provide basic information about your country and provide the date and reference or basis for your answers Population Incidence of diabetes Incidence of diabetic foot (infections/ Incidence of foot amputations? Incidence of diabetic foot amputations? Incidence of minor foot amputations (toe/forefoot)? Incidence of major amputations (BKA/AKA) Number of general surgeons Number of orthopedic surgeons Number of podiatrists Number of endocrinologists Number of infectious disease specialists Number of vascular surgeons Number of wound care nurses Number of Wound care specialists or others with diabetic foot interest. If not listed identify their profession.

The responses were compiled in a Microsoft Excel database. Descriptive statistics were used to report the numbers and percentages (%) of responses. Since no individual or identifiable patient information was requested, no consent was required. This multi-national survey was waivered from Ethic Board approval.

## Results

Responses from 25 countries (100%) were obtained (Table 2). 81% of respondents could not identify any MOH, Hospital or individual initiatives that monitored DF. Only 9 (36%) countries had guidelines for the management of the DF and they were distributed by different agencies. The main source appeared to be the National Diabetes Association or Medical Association. The MOH was only rarely responsible for disseminating the guidelines. The protocols were generally adapted from existing international guidelines, especially by the International Diabetes Foundation (IDF) but some countries developed their own. Only 3 countries utilized IWGDF guidelines.

**Table 2:** 

Questions	YES
Do you know if your country has guidelines for the management of the diabetic foot?	9 (36%)
Could you provide it?	9 (36%)
Does your country have a Diabetes Association	11 (44%)
Do they have guidelines for the management of the diabetic foot?	3 (13%)
Does your country have a representative on the DFoot International?	5 (20%)
Does your country have a Medical (or other Paramedical) Association?	11 (44%)
Do they have guidelines for the management of the diabetic foot?	0
Does your country have a University Department of Surgery/Medicine /Primary Care?	10 (40%)
Do they have guidelines for the management of the diabetic foot?	5 (20%)
Does your country have a Ministry of Health?	11 (44%)
Is there a designated individual that deals with diabetes and/or diabetic foot?	5 (20%)
Do they have guidelines for the management of the diabetic foot?	1 (4%)
Please provide basic information about your country and provide the date and reference or basis for your answers e. Number of general surgeons f. Number of orthopedic surgeons g. Number of podiatrists h. Number of endocrinologists i. Number of infectious disease specialists j. Number of vascular surgeons k. Number of wound care nurses l. Number of Wound care specialists or others with diabetic foot interest.	25 (100%) 10 (40%) 6 (24%) 5 (20%) 2 (8%) 10 (40%) 16 (64%) 4 (16%)

There appeared to be a fair number of general and orthopedic surgeons on the islands, but there were scarce or absent podiatrists, endocrinologists, vascular surgeons, or infectious disease specialists. Only 6 (24%) countries had podiatrists and 10 (40%) had vascular surgery availability but only three countries with specialist vascular training. In 4 out of the 10 countries, although vascular surgery was available, it was not lower limb focused. 7 (28%) countries had the components for a multidisciplinary clinical team, but we could not identify any that had a functional unit.

Out of those countries that participated only 2 respondents had definitive data on the incidence of diabetic foot infection or ulceration; 3 knew the incidence of diabetic foot amputations, none knew the incidence of minor foot amputations and only 6 knew the incidence of major amputations.

## Discussion

The high prevalence of DF disease and LE amputations in the Caribbean has been long recognized [5, 19] but, unfortunately, very little progress has been made despite reports of improved outcomes around the world [13, 14]. The reasons underlying this lack of progress are not clear. This study was undertaken to determine whether the Caribbean countries have designated individuals or organizations that monitor DF disease and whether there are DF protocols consistent with the IWGDF guidance documents, and whether they are implemented by the health institutions, MOH or medical training facilities. Our study revealed some interesting observations. 20 of the 25 countries (80%) were members of the IDF and yet, only 5 countries (20%) had any guidance documents, and these were primarily managed by either interested professionals, the Diabetes Association or the Medical Association. Only 2 countries used the IWGDF guidance documents. Surprisingly, it did not appear that the MOH was involved. In most cases the MOH was unaware of guidelines for the DF. Second, there was a dearth of information regarding the incidence of DF disease and the number of major and minor amputations. There was little awareness of the scope of the DF problem. This is despite published data available (Table 3) [20].

**Table 3:** 

COUNTRY	Population	DM Incidence
**Anguilla** **	9,670	13.3
**Antigua**	69,700	13.3
**Aruba (D)**	77,800	15.0
Bahamas	393,244	9.2
**Barbados**	204,100	17.8
**Belize**	229,300	14.9
**Bermuda** **	43,400	15.8
Bonaire (D)	20,104	17.8
**British Virgin Islands**	21,100	14.7
**Cayman Islands** **	41,400	14.2
**Curacao (D)**	116,000	17.0
**Dominica**	48,900	12.9
**Dominican Republic**	6,656,000	8.7
**Grenada**	69,500	9.7
**Guyana**	479,100	10.5
**Haiti**	6,396,200	5.7
**Jamaica**	1,936,500	11.7
**Montserrat**	3,400	13.9
**St. Kits and Nevis**	36,900	14.2
**St. Lucia**	129,300	11.5
**St. Maarten (D)**	26,800	14.2
St. Vincent’s	110,940	11.6
**Surinam**	367,800	13.0
**Trinidad & Tobago**	982,600	12.3
Turks & Caicos**	38,718	11.0

BOLD = IDF member (n = 21).

** Associate Member CARICOM (n = 5).

Member CARICOM (n = 15).

(D) Dutch Caribbean (n = 4).

TOTAL 25 countries.

https://idf.org/our-network/regions-members/north-america-and-caribbean/members.html [20]

The survey demonstrated that although the respondents acknowledged there was a high incidence of diabetes, DF infections or LE amputations in their country, there was a general lack of specific information that correlated with published data. Regarding the presence of a multidisciplinary DF team, there was also a wide disparity among the countries. Most countries have mainly general surgeons and a few orthopedic surgeons. Only a quarter have a podiatrist and under half had availability of vascular surgery, most of whom were not specialty trained. Only 3 countries reported specialty trained plastic surgeons that might have the skills of performing foot reconstructions for limb salvage. In contrast, the Dominican Republic reported over 200 general surgeons and orthopedic surgeons, 150 endocrinologists, 70 infectious disease specialists and 10 vascular surgeons but no podiatrist. Based on our study, it is clear that there are scant DF interdisciplinary teams in the Caribbean.

Obviously, no clear answer exists regarding which providers should be involved in this team approach or the extent of involvement provided by each member. It is well-established that an aggressive interdisciplinary approach to DF disease is required to provide optimal medical and surgical care for improved outcomes [13]. The presence of multiple practitioners caring for the same patient increases the opportunity for life-long follow-up surveillance of vascular and podiatric disease [13]. Numerous centers around the world have reported significant reductions in amputations and ulcer recurrence when limb assessment protocols have been established and an interdisciplinary team assembled [14, 21]. It is understandable that there are many barriers to forming a multidisciplinary team and establishing the right support structure for it to become successful. Our survey confirms that there are no regional centers in the Caribbean, to work towards this process of IWGDF implementation strategies which are foundational for the success of DF disease management. It is possible to assume that there is an inert disunity, within the countries that stave away team building capacity, for limb salvage.

Regardless, it is clear that limb preservation requires a series of steps including re-establishing adequate perfusion through adequate investigations inclusive of the microvascular status which is often overlooked, serial wound debridements, appropriate wound managed care and accessibility to materials to encourage prompt wound healing, aggressive infection management, and correction of underlying biomechanical abnormalities [1, 12, 13]. At a minimum, vascular surgeons with specialty in the lower limb, podiatrists and podiatric surgeons are essential components of the team [13]. Optimized wound care is then critical after required medical and surgical interventions have been accomplished. Whilst preventative DF care has a key role in managing the DF, it is essentially as important that when assaults to the DF occur the entire team is engaged whether under the same roof, within the same country or across the waters based on limited human specialty resources, as whatever is deemed necessary for saving a limb. Primary care physicians and podiatrists play important gate-keeper roles in monitoring DF and managing early foot trauma and infections.

Unfortunately, not all critical components of an interdisciplinary team are available in either general hospitals or wound care facilities in the Caribbean [11]. Some individual physicians and surgeons with experience and training across a broad spectrum of disciplines may appropriately treat conditions in areas that lack dedicated limb preservation centers, but for complex cases, the limb salvage results will likely be inferior to the team approach. Therefore, while the constituents of teams may differ in various locales based on myriad factors, there are certain critical elements in the management of a DF that constitutes an essential, professional skill set required of a dedicated DF care team. There are some bright spots in the Caribbean. Guyana reported a dramatic improvement in LE amputations with utilization of a complex interprofessional team and foot care based protocol [21–23]. Health care workers were trained with Canadian based programs including the International Interprofessional Wound Care Course (IIWCC) Michener Institute Diabetes Educator course, regionalized to cover approximately 90% of the population. Over a period of 5 years, there was an approximately 70% rate reduction in LE amputations. This government-initiated project, backed by dedicated medical staff has continued to this day and has been touted as a vision for all other Caribbean territories to emulate. Unfortunately, this country is the exception and most of the governments of the Caribbean and their local surgical communities do not have the capacity to establish and sustain the kinds of teams that are so desirable.

Given that diabetes is noted not only as the most common non-communicable disease (NCDs), but also as a leading cause of death due to its myriad of complications which can also lead to disability [27], it is perplexing that greater resources are not allocated to proactive mechanisms to stamp out such suffering. For a dire condition of over 50 years of academia and research on the diabetic foot, it was disappointing to note lack of knowledge of guidance protocols. Does this show that the disease, because it is so common, has made our health professionals become immune to this condition? Or does it exhibit the perpetual spiral of clinical inertia to implement programs and multidisciplinary teams? Or does it show the general lack of understanding of what a multidisciplinary team requires within the region? In either case it is a low hanging fruit given the expertise available to this region. It could signify that there was little priority placed on the DF, protocols for DF or LE amputation prevalence. It also possibly highlights the frustration at the complexity of the management of DF disease itself and/or general apathy towards the DF problem. Much of this may be due to the public’s perception of doctors and hospitals, in what might be termed the “amputation cycle”. As citizens are made aware of a countries high-rate of LE amputations they become hesitant to see their physicians at the early-stages of their lower limb disease believing that they too might be at risk of an amputation. Moreover, there are also potential national and physician factors relevant to this situation. The existence of a “substitution culture” [15] and compliance issues [24] transcends the Caribbean.

Our study has some nuances and limitations. First, although we were primarily interested in the Caribbean nations, we queried the CARICOM countries which includes some South and Central American countries (Guyana, Suriname, and Belize) because of their strong economic, political, and social linkages [2]. We also included the Dutch Caribbean islands (Aruba, Bonaire, Curacao and St. Marteen) and the Dominican Republic because of their strong ties to the neighboring island nations. The Caribbean countries are not only geographically diverse, being separated by the Caribbean Sea, but are a mix of races, with predominant African ancestry and a heavy mix of Indian, Chinese, and European backgrounds. The racial admixture varies between countries and may account for some of the demographic disparities within the Caribbean nations [25]. For example, the rates of amputation are higher in Afro-Trinidadians compared to Indo-Trinidadians [26]. Therefore, Caribbean countries may place different priorities on this problem.

Second, we did not include the US Virgin Islands or Cuba because of their distinct health economies. Cuba is one of the 20 countries of the IDF SACA region and the prevalence of diabetes in adults is 13.2%. The Cuban government operates a national health system and assumes fiscal and administrative responsibility for the health care of all its citizens and is unlike the neighboring Caribbean countries. The US territories also have a distinct health economics. The Virgin Islands have universal healthcare and have a law that states that the hospitals cannot deny benefits or services because of a person’s inability to pay. Therefore, if it’s unavailable on the Virgin Islands, the hospitals must be accommodated on the mainland of the USA. This makes the management of the diabetic foot unparallel to the other Caribbean territories.

Third, it is also interesting to note that only 3 countries (Haiti, Dominican Republic and Grenada) had reported diabetes mellitus incidence rates less than 10% (Table 3). However, these statistics may reflect lack of population testing, reporting or poor access to healthcare by the population. For instance, a study of Haitians living in Miami revealed a diabetes prevalence rate of 33% [27].

Lastly, the respondents to our questionnaire were DF health care personnel who represented different sectors of the nations. This underscores one of the primary problems of the island nations in the lack of any consistent group that was responsible for overseeing DF education and management. We made an extensive effort to contact individuals starting with a hierarchy of the MOH, the Diabetic Association, the Medical Association, medical schools, government and private hospitals and clinics along with aligning their published memberships or affiliation to IWGDF. The MOH was, for the most part, almost oblivious to the impact of the DF to the citizen’s health and so reliance on the management of the DF was left to the national diabetes associations or national medical associations. Unfortunately, as demonstrated by our study, even these organizations had inconsistent implementation of national or IWGDF guidelines.

Taken as a whole, our study demonstrates what appears to be a lack of a conscientious systematic approach by Caribbean developing countries to DF disease. DF disease seems to be managed ad hoc, and based on past experiences, memories and perceptions as opposed to scientifically known evidence-based practices. The lack of implementation of such approaches can be seen to have caused both a delayed response to seek help for the DF patient and an impression of a frustrated approach on the part of local treating physicians. Based on evidence-based approaches in the international arena, there are confirmations of the value of interdisciplinary approaches to managing the diabetic foot and promotion of avoidable amputations [13, 14, 21]. Government and MOH support are crucial for success and to help ease some of the barriers mentioned. In addition to health care interaction, dedicated infrastructure such as dedicated community foot clinics, vascular laboratory, endovascular operating rooms are necessary along with the budget for consumables and wound products.

In 1989, the St. Vincent Declaration proposed an aggressive approach to diabetes-related complications to reduce DF complications and LE amputations [28]. The recommendation for the establishment of vascular units was advised to reduce the amputation rate. However, our study indicated that only 10 countries had vascular specialist availability and only 4 countries had trained specialists with a dedicated unit. Another initiative in 2009 was the “Step-by-step” program of the World Diabetes Foundation spearheaded by and funded jointly through the Rotary Clubs [29]. Although the mission was for the health care teams to educate diabetes patients and the general population about preventive measures for DF problems and facilitate development of algorithms for foot care to enable and encourage multidisciplinary teamwork and unify diabetes care services, there has been no continuity or guidelines put in place for a teamed approach.

The role of primary care physicians and podiatrists in performing annual foot examinations to identify high-risk foot conditions such as neuropathy, vascular disease and foot deformities cannot be over-emphasized. Collaborative interaction among other diabetes care givers is optimal to provide patients with glycemic control, smoking cessation and patient education on daily foot care and use of proper footwear. In countries without a multidisciplinary team or with no input from the vascular or podiatric team., a significant number of proximal LE amputations are done as primary procedures [6, 13]. Appropriate, timely patient referral and dedicated service for the management of foot wounds and DF infection is crucial for improved outcomes. Input from the interdisciplinary team is critical. The need for DF guidelines and programs in the Caribbean is critical and mandatory at this stage.

In conclusion, it is expected that with reported improved outcomes of interdisciplinary approaches to the DF, there will be a motivation to have changed behavior of patients presenting early and physicians gaining knowledge of how such referrals can be appropriately guided to ensure preservation of a functional foot. Given the persistent trends of over 50 years of LE amputations, it is highly recommended, using developed country baseline results for successes with limb salvage, that the MOH, and relevant institutions consider implementation of multidisciplinary DF teams, DF guidance protocols and/or programs through policies which will enhance the streamlining of the at-risk DF, and screening programs to prevent DF ulcerations. Despite previous efforts for assisting MOH, there has been a lack of continuity. Therefore, there is also a need for MOH to actively facilitate a gatekeeper for continuity of these programs. Without the framework in place to facilitate implementation, it is expected that the revolving door feeding the frustrations and apathetic approach to the DF and the ensuing high rate of LE amputations will continue.
